# Childhood Adversity and Natural Disaster Exposure Interact to Impact Older Adult Cognitive Functioning

**DOI:** 10.1093/geroni/igaf122.2280

**Published:** 2025-12-31

**Authors:** Karen Lawrence, Yeon Jin Choi

**Affiliations:** University of Kentucky, Lexington, Kentucky, United States; University of Kentucky, Lexington, Kentucky, United States

## Abstract

The frequency and severity of natural disasters are increasing due to climate change; these events may be perceived as stressful or traumatic. Studies have shown that childhood trauma is associated with poorer cognitive function in older adults yet research on the impact of natural disasters on this relationship is rare. Leveraging data from the Health and Retirement Study, we investigated whether having experienced a natural disaster moderates the relationship between childhood adversity (range: 0–4 adversities) and cognitive function in 12,995 adults 50+ years of age [Mage=64.6, SD = 10.1]. Test scores from total word recall, serial 7’s test, and backwards counting were summed to calculate an overall cognitive function score (range: 0-27). At least one childhood adversity was experienced by 35.6% and a natural disaster by 17.9% of the sample. In a weighted regression model, adjusted for sociodemographic characteristics and health conditions, the interaction between childhood adversity and natural disaster exposure was associated with .25 points lower cognitive function score [β = -.25, 95%CI: -.45, -.05]. Respondents with childhood adversity had significantly lower cognitive function than those without, when exposed to a natural disaster. Among those who experienced a natural disaster, the estimated cognitive function score was 15.56 for those without childhood adversity. Those who experienced one, two, three, or four adversities had lower cognitive function at 15.17, 14.78, 14.39, and 14.00, respectively. These findings suggest that natural disaster exposure is a potential environmental stressor that can exacerbate the effects of childhood trauma on cognitive function outcomes later in life.

